# Patient pain during intravitreal injections under topical anesthesia: a systematic review

**DOI:** 10.1186/s40942-017-0076-9

**Published:** 2017-07-03

**Authors:** Helio Francisco Shiroma, Augusto Key Karazawa Takaschima, Michel Eid Farah, Ana Luisa Höfling-Lima, Graziela de Luca Canto, Roberto Henrique Benedetti, Eduardo Buchele Rodrigues

**Affiliations:** 1 Department of Ophthalmology and Visual Sciences, Federal University of São Paulo, Rua Pastor William Richard Schisler 900/apto 1011, Florianópolis, SC 88034-100 Brazil; 2 Florianopolis Hospital, Florianópolis, Brazil; 3 Brazilian Centre for Evidence-based Research, Health Sciences Centre, Federal University of Santa Catarina, Florianopolis, Brazil; 4 Unisul—Universidade do Sul de Santa Catarina, Tubarão, Brazil

## Abstract

**Background:**

Intravitreal injection (IVI) is a very common vitreoretinal procedure, and multiple injections are often required per patient. This systematic review was conducted to evaluate the effectiveness of various local anesthetic techniques in reducing pain during injection.

**Methods:**

A systematic review was conducted based on searches of Cochrane, LILACS, PubMed, Scopus, Web of Science, and the gray literature (Google Scholar). The end search date was February 19, 2016, across all databases. We classified pain by converting visual analog scale (VAS) scores (0–100 mm) into Jensen’s classification levels: 0–4, no pain; 5–44, mild pain; 45–74, moderate pain; and 75–100, severe pain. An intervention was considered clinically significant when pain score change was >12 mm on a 100-mm scale.

**Results:**

Eight studies out of 23 met the eligibility criteria. The total number of patients was 847. Most studies (5/8 [62.5%]) were at unclear risk of bias because of unclear randomization, thus providing only moderate evidence to this review. The anesthetic techniques included eye drops with proparacaine, tetracaine or cocaine, a lidocaine pledget or gel, and subconjunctival injection of 2% lidocaine or 0.75% levobupivacaine. No study comprised all of the techniques. Pain was mild (VAS scores, 5–44 mm) regardless of anesthetic technique. A clinically significant intervention (pain score change >12 mm) was found for only one study comparing proparacaine drops, lidocaine gel, and subconjunctival lidocaine; in that study, a subconjunctival injection of 2% lidocaine provided the greatest pain reduction. A meta-analysis was not possible due to study heterogeneity.

**Conclusions:**

Patient pain during IVI under topical anesthesia is mild regardless of anesthetic technique. A subconjunctival injection of 2% lidocaine could be an option for highly sensitive patients. However, with moderate level of evidence, no single anesthetic technique could be defined as the best option for IVI.

**Electronic supplementary material:**

The online version of this article (doi:10.1186/s40942-017-0076-9) contains supplementary material, which is available to authorized users.

## Background

Intravitreal injection (IVI) is one of the most common vitreoretinal procedures performed nowadays [1]. Steroids, antibiotics, and antiviral drugs have been injected into the vitreous humor for many years, but the use of IVI has increased dramatically only recently after the introduction of anti-vascular endothelial growth factor (VEGF) therapy for neovascular age-related macular degeneration [2]. Other indications for IVI of anti-VEGF include diabetic retinopathy, vascular occlusions, and cystoid macular edema [3].

Patients may experience pain during IVI [3], especially if multiple injections are required. The use of local anesthetics minimizes pain and avoids intraocular complications caused by pain-induced rapid, uncontrolled movements of the eye [4]. Based on recent surveys, most ophthalmologists (65–90%) perform IVI using local anesthetic eye drops [5]. Other techniques include the use of an anesthetic gel, peribulbar block, subconjunctival injection, and a pledget soaked in anesthetic [3]. However, there is no consensus on the best anesthetic option [6].

The choice of a single anesthetic technique for IVI requires careful evaluation of patient pain, ideally using an objective measure [7]. However, patient pain is typically evaluated using one-dimensional tools such as numeric rating or visual analog scales [8]. Such type of scales raises concerns of bias [9].

To our knowledge, no systematic review has addressed patient pain during IVI under topical anesthesia. The aim of this systematic review was to evaluate the effectiveness of different local anesthetic techniques for IVI within the limitations of analog (visual or oral) pain scales.

## Methods

This systematic review was written in accordance with the Preferred Reporting Items for Systematic Reviews and Meta-Analyses (PRISMA) checklist [10].

### Protocol and registration

The systematic review protocol was registered at the International Prospective Register of Systematic Reviews (PROSPERO) under number CRD42016037099.

### Terminology

For this systematic review, we compared different techniques of local anesthesia for IVI of antiangiogenic agents and steroids. Antiangiogenic agents included bevacizumab (Avastin^®^; Genentech/Roche, USA), ranibizumab (Lucentis^®^; Novartis, Switzerland), and aflibercept (Eylea^®^; Bayer HealthCare, Germany). Steroids included biodegradable dexamethasone implant of sustained release and triamcinolone. Local anesthetics included eye drop anesthetics (tetracaine, proparacaine, and cocaine), lidocaine gel, lidocaine pledget, subconjunctival anesthesia, and peribulbar block. Anesthetic and/or analgesic effect was evaluated using analogue (visual or oral) pain scales with grades ranging from 0 to 100 mm or from 0 to 10 cm.

### Study design

The aim of this systematic review was to evaluate the effectiveness of different local anesthetic techniques for IVI of anti-VEFG agents or steroids within the limitations of analogue pain scales. Studies were selected in two steps. First, we classified patient-reported pain scores for each anesthetic technique covered by individual studies. Scales ranging from 0 to 10 cm or points were converted to a range from 0 to 100 mm. Other scales were not included in this review, due to heterogeneity. We classified pain by converting visual analog scale (VAS) scores (0–100 mm) into Jensen’s classification levels [8]: 0–4, no pain; 5–44, mild pain; 45–74, moderate pain; and 75–100, severe pain. Second, we considered that an intervention was clinically significant when pain score change was >12 mm on a 100-mm scale. The relative value difference with clinical significance in patient perception comparing different treatments for pain varies from 9 to 15 mm in a 0 to 100 mm scale [9, 11], but higher values have been reported [12]. For a conservative approach, we chose the average value of 12 mm, which we think reflects a clinically meaningful difference in pain perception.

### Information sources

Studies to be considered for inclusion were identified by searching the following electronic bibliographic databases: Cochrane, LILACS, PubMed, Scopus, and Web of Science. An additional search of the gray literature was performed using Google Scholar. The end search date was February 19, 2016, across all databases. In addition, the reference lists of the selected articles were searched manually.

Appropriate truncation and word combinations were selected and adapted for each database search (Additional file 1: Table S1), with the aid of a health sciences librarian. All references were run through the reference manager software Mendeley^®^ (Elsevier), and duplicate hits were removed.

### Study selection and eligibility criteria

We reviewed studies whose objective was to compare the effect of different local anesthetic techniques for IVI of antiangiogenic agents and steroids using an analog (visual or oral) pain scale ranging from 0 to 100 (or 0–10).

There were two phases of review. In phase 1, we reviewed titles and abstracts and excluded the following: (1) studies conducted in infants (0–18 years); and (2) reviews, letters, conference abstracts, and editorials. In phase 2, we reviewed full-text articles and additionally excluded the following: (3) studies including sedation or general anesthetics for IVI; (4) studies including IVI of medications other than antiangiogenic agents or steroids; (5) non-randomized clinical trials; (6) studies using a pain scale other than an analogue (visual or oral) scale ranging from 0 to 100 or 0 to 10; (7) studies not evaluating pain at the moment of injection; and (8) studies not using mean as the measure of central tendency for pain score.

Two authors (HS, AT) independently reviewed all search results. In both phases, when disagreements emerged between the two reviewers, they tried to reach a consensus. When they were unable to reach a consensus, a third author (GLC) made the final decision. Articles that did not appear to meet the inclusion criteria were discarded. In phase 2, the same authors reviewed the full-text of the articles. The third author (GLC) read the abstracts of all the selected articles and made the final decision on inclusion; however, final selection was always based on the full text of the publication. The reference lists of selected studies were critically assessed by both HS and AT.

### Data collection

One author (HS) collected data from the selected studies. The following information was recorded: study background (authors, year, country, study design, and objective), population characteristics (number of patients, mean age), interventions (anesthetic techniques, type of medication, and pain grading), and outcomes (average pain score at the moment of injection and main conclusion). A second author (AT) crosschecked all the collected information and confirmed its accuracy. Again, any disagreement was resolved by discussion and mutual agreement among the three reviewers (HS, AT, LC).

### Risk of bias in individual studies

The methodology of the selected studies was evaluated using the Cochrane Collaboration’s risk of bias tool [13]. The following characteristics were included in the assessment: sequence generation, allocation concealment, blinding of participants and personnel, potential threats to validity of performance, blinding of outcome assessment, and potential threats to validity of detection of bias [13]. Two reviewers (HS, AT) independently assessed the quality of each included study. Disagreements between the reviewers were resolved through discussion.

### Outcome measures

The main outcome measure was the evaluation of the anesthetic/analgesic effect of different anesthetic techniques for IVI using analog (verbal or visual) pain scales.

### Synthesis of results and risk of bias across studies

If feasible, the possibility of meta-analysis and risk of bias across studies was considered.

## Results

### Study selection

**Fig. 1:**
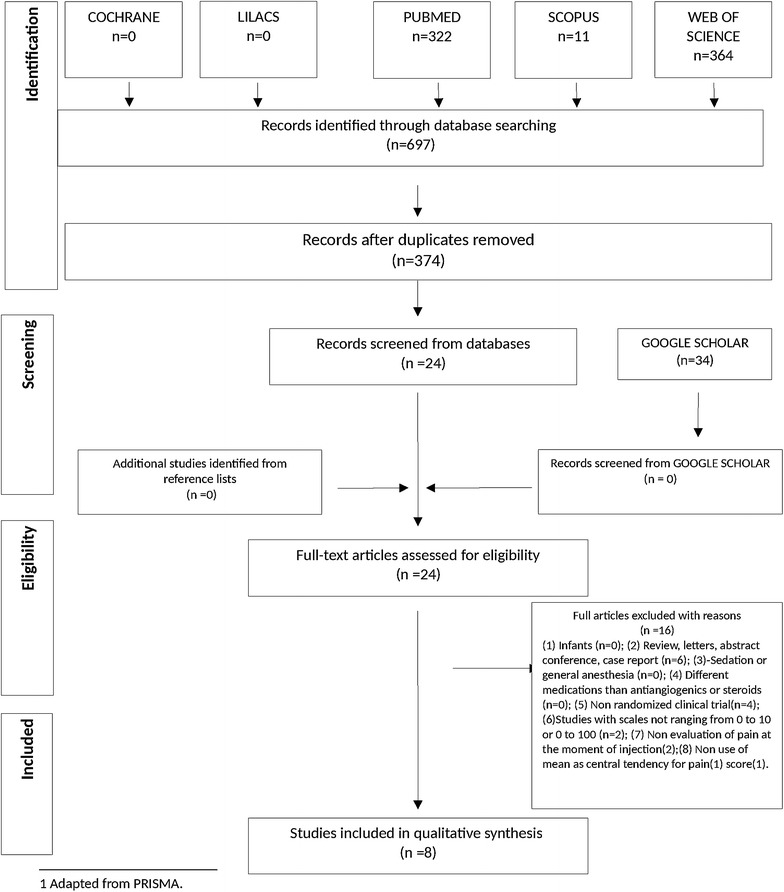
Flow Diagram of literature search and selection criteria. (adapted from PRISMA)

During the initial search (phase 1) and following duplicate removal, 374 different citations were identified across the five electronic databases. An additional search using Google Scholar found no additional relevant articles. After a comprehensive evaluation of the abstracts, 23 articles were deemed potentially relevant and were selected for phase 2. Of these 23 studies selected in the first evaluation, two studies using biodegradable dexamethasone implant included. However, those two studies were excluded, because they were not randomized. Others 13 were excluded (Additional file 2: Table S2). Thus, only eight studies were retained for the final selection. A flowchart of the process of literature search and selection is shown in Fig. 1.

### Study characteristics

A summary of the study characteristics is shown in Table 1. The selected studies were conducted in Brazil [1, 6], Canada [11], Turkey [14], UK [15], and USA [2, 5, 16] from 2008 to 2015. The total number of patients for all studies was 847. The injected medications were ranibizumab, bevacizumab, and triamcinolone.

**Table 1:** Summary of characteristics of the included studies

Study background		Participants		Interventions			Outcomes	
References (country)	Objective	Cases, *n*	Mean age, years	Groups according to anesthetic technique	Pain grading	Drug	Average pain score	Main conclusion
Blaha et al. [16] (USA)	To compare pain scores during injection versus total procedure for four anesthetic techniques	96	80	G1: 0.5% topical proparacaine	Pain was graded on a 0 to 10 scale	RNZ, BVZ	G1: 3.0	No statistical difference in pain scores for the four anesthetic techniques. Topical drops provided excellent anesthesia in a quick, comfortable, and safe manner for IVI
				G2: 0.5% topical tetracaine			G2: 2.8	
				G3: topical proparacaine + 4% lidocaine pledget			G3: 2.3	
				G4: topical proparacaine + SC injection of 2% lidocaine			G4: 3.1 *p* = 0.28	
Yau et al. [11] (Canada)	To compare the anesthetic effectiveness of three topical agents used for IVIs	93	Group 1: 83.6	G1: 0.5% tetracaine hydrochloride drops + 4% lidocaine pledget for 10	Patients graded pain on a 100-mm VAS, or by saying a number from 0 to 100	RNZ	G1: 19 (12–26)	No clinical (statistical) difference in patient pain between the three topical agents tested. The addition of a 4% lidocaine pledget for 10” offered no clinical advantage in pain relief compared to 0.5% tetracaine or 4% cocaine drops alone
			Group 2: 79.5	G2: 0.5% tetracaine hydrochloride drops			G2: 21 (13–29)	
			Group 3: 82.1	G3: 4% cocaine + epinephrine 1/100.000 drops			G3: 21 (16–27)	
Örnek et al. [14] (Turkey)	To compare the efficacy of topical 0.75% levobupivacaine and 0.5% proparacaine	96	63.97	G1: 0.75% levobupivacaine drops	Patients graded pain on a 100-mm VAS	RNZ, TAC	G1: 44.77 ± 16.42	0.5% topical proparacaine was more effective than 0.75% topical levobupivacaine in preventing pain during IVI
				G2: 0.5% proparacaine drops			G2: 34.18 ± 14.83 *p* = 0.003	
Shiroma et al. [1] (Brazil)	To investigate the safety and anesthetic efficacy of five concentrations of lidocaine gel	260	70.1	G1: 2% lidocaine gel	Patients graded pain on a 0 to 10 scale	RNZ	G1: 2.63 ± 1.68	Lidocaine gel at concentrations from 2% to 12% induced similar anesthetic effect for IVI
				G2: 3.5% lidocaine gel			G2: 2.08 ± 1.35	
				G3: 5% lidocaine gel			G3: 2.00 ± 1.65	
				G4: 8% lidocaine gel			G4: 1.93 ± 1.40	
				G5: 12% lidocaine gel			G5: 1.83 ± 1.35 *p* = 0.077	
Andrade et al. [6] (Brazil)	To compare the anesthetic effectiveness of topical proparacaine drops, SC lidocaine, and 2% lidocaine gel	92	66.4	G1: proparacaine	Patients graded pain on a 0 to 10 scale	BVZ	G1: 3.2 ± 1.7	SC injection of lidocaine was most effective in preventing pain during IVI compared to proparacaine or 2% lidocaine gel
				G2: proparacaine + SC injection of 2% lidocaine			G2: 1.0 ± 1.0	
				G3: 2% lidocaine gel			G3: 1.0 ± 1.1	
Kumar et al. [15] (UK)	To compare patient comfort during IVI after SC anesthesia or topical eye drops	30	G1: 72	G1: 0.5% proximetacaine	Patients graded pain on a 0 to 10 scale	TAC	G1: 0.87 ± 0.83	There was no significant difference in pain scores or overall satisfaction scores between the two groups
			G2: 74	G2: SC injection of 2% lidocaine			G2: 0.93 ± 0.96 *p* = 0.84	
Davis et al. [2] (USA)	To compare the anesthetic effect among topical proparacaine drops, 4% lidocaine solution, and 3.5% lidocaine gel	120	80.18	G1: 0.5% topical proparacaine	Patients graded pain on a 0 to 10 scale	BVZ, RNZ, TAC	G1: 1.78 ± 1.44	There was no significant difference in pain scores or overall satisfaction scores between the different groups
				G2: topical proparacaine + 4% lidocaine pledget			G2: 1.75 ± 1.46	
				G3: 3.5% lidocaine gel			G3: 1.48 ± 1.58 *p* = 0.38	
Rifkin and Schaal [5] (USA)	To determine factors associated with patient comfort during in-office IVI	60	65	G1: 0.5% TetraVisc	Patients graded pain on a 0 to 10 scale	BVZ, RNZ, TAC	G1: 3.39 ± 2.26	The tetracaine group reported the lowest pain
				G2: proparacaine			G2: 3.17 ± 2.18	
				G3: tetracaine			G3: 3.05 ± 2.01 *p* < 0.01	

*G* group, *IVI* intravitreal injection, *SC*, subconjunctival, *RNZ*, ranibizumab, *BVZ* bevacizumab, *AFL* aflibercept, *TAC* triamcinolone acetonide

### Risk of bias within studies

**Fig. 2:**
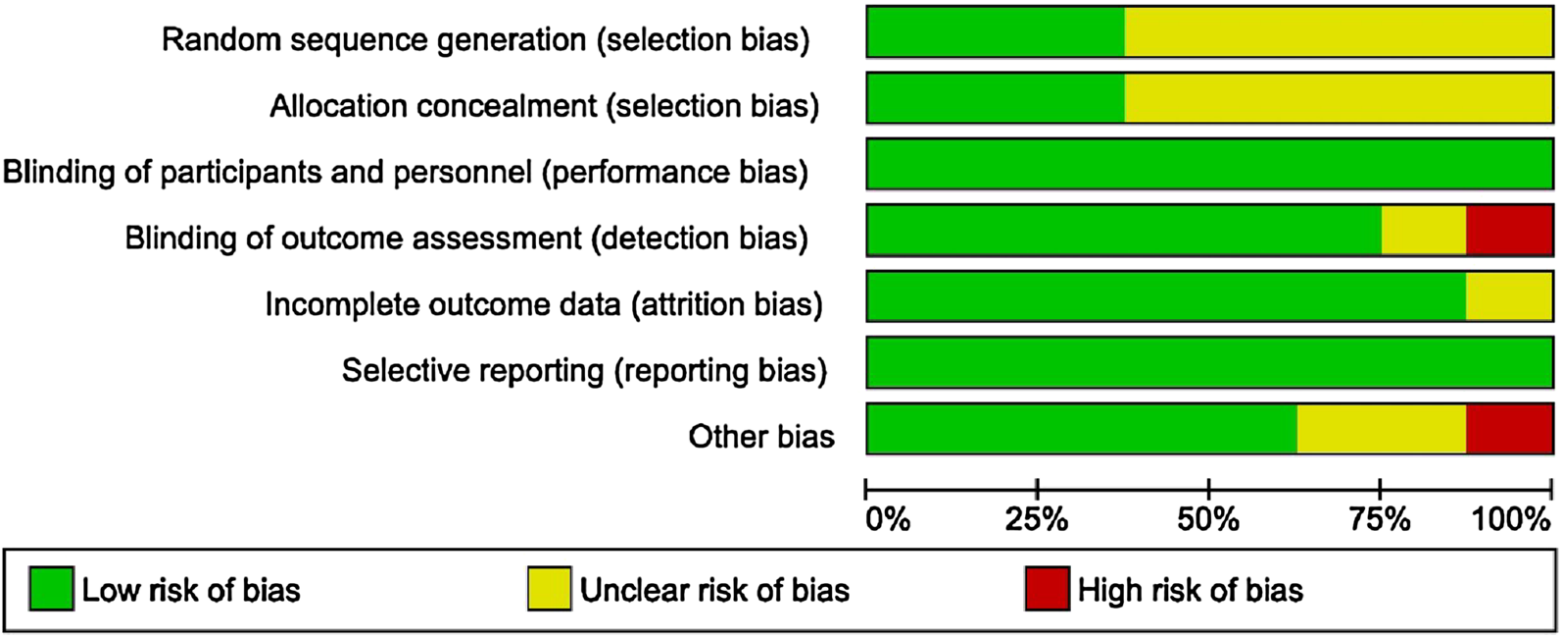
Risk of bias graph: review authors’ judgments about each risk of bias item presented as percentages across all included studies (n = 8)

**Fig. 3:**
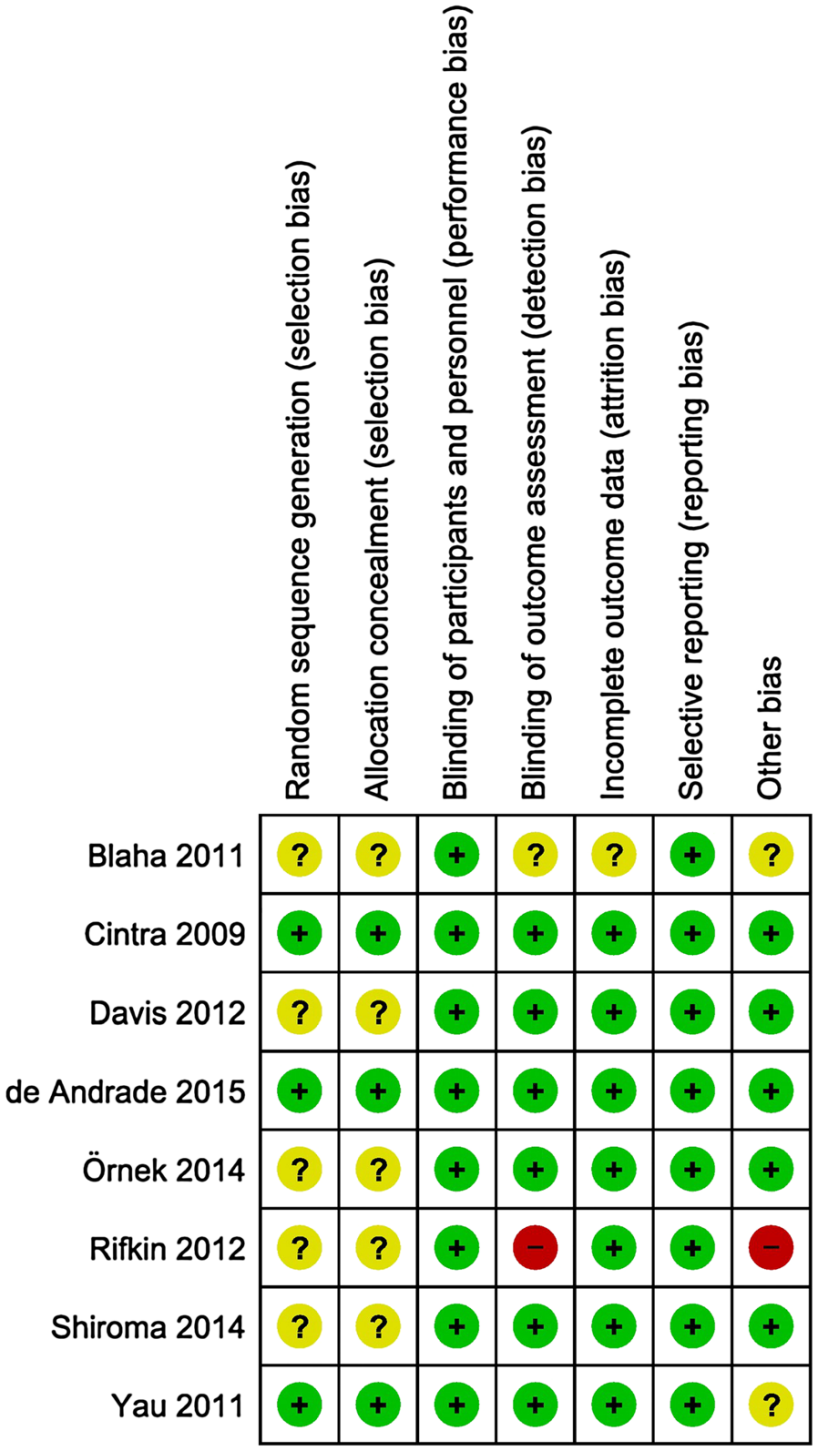
Risk of bias summary: review authors’ judgments about each risk of bias for each included study

Selection biases were evaluated through random sequence generation and allocation concealment. We judged only three (37.5%) of the eight studies to be at low risk of bias based on random sequence generation and allocation concealment; the other studies were considered to be at unclear risk of bias because of unclear randomization process, thus providing moderate level of evidence to this review. We present other potential sources of bias as percentages in Fig. 2 and per study in Fig. 3.

### Characteristics of included studies

The characteristics of the eight studies evaluating the effect of different local anesthetic techniques for IVI of antiangiogenic agents and steroids using analog (visual or oral) pain scales are summarized in Table 1.

### Anesthetic methods

Different anesthetics methods were recorded and compared among the included studies. The described techniques included eye drops with proparacaine [2, 5, 6, 14–16] and tetracaine [5, 11, 16], 4% lidocaine pledgets [11], 4% cocaine + epinephrine 1/100,000 drops [11], subconjunctival injection of 2% lidocaine [6, 15], lidocaine gel at 2, 3.5, 5, 8 and 12% [1, 2, 6], and 0.75% levobupivacaine [14].

### Classification of studies according to pain

We classified pain by converting (VAS) scores (0–100 mm) into Jensen’s classification levels [8]: 0–4, no pain; 5–44, mild pain; 45–74, moderate pain; and 75–100, severe pain. Only six studies evaluated pain at the moment of IVI [1, 2, 5, 6, 11, 14]. On the other hand, Blaha et al. [16] and Kumar et al. [15] considered a combined score calculated by adding the discomfort for anesthesia and the IVI.

Patient pain was mild in all studies regardless of anesthetic technique or definition of pain scores (single or combined) (Table 2). A meta-analysis was not possible due to high study heterogeneity.

**Table 2:** Pain in the included studies according to Jensen’s classification [8]

References (country)	Groups according to anesthetic method	Difference in pain	Mean pain score related to discomfort of anesthesia	Mean pain score during injection	Mean global pain	Jensen’s classification
Blaha et al. [16] (USA)	G1: 0.5% topical proparacaine	NS	G1: 14	G1: 30	G1: 44	G1: MI
	G2: 0.5% topical tetracaine		G2: 7	G2: 28	G2: 35	G2: MI
	G3: topical proparacaine + 4% lidocaine pledget		G3: 16	G3: 23	G3: 39	G3: MI
	G4: topical proparacaine + SC injection of 2% lidocaine		G4: 10 *p* = 0.17 (NS)	G4: 31 *p* = 0.28 (NS)	G4: 41 *p* = 0.65 (NS)	G4: MI
Yau et al. [11] (Canada)	G1: 0.5% tetracaine hydrochloride drops + 4% lidocaine pledget for 10	NS	NA	G1: 19	NA	G1: MI
	G2: 0.5% tetracaine hydrochloride drops			G2: 21		G2: MI
	G3: 4% cocaine + epinephrine 1/100.000 drops			G3: 21		G3: MI
Örnek et al. [14] (Turkey)	G1: 0.75% levobupivacaine drops	Statistically different, but not clinically significant	NA	G1: 44.77	NA	G1: MI
	G2: 0.5% proparacaine drops			G2: 34.18 *p* = 0.003		G2: MI
Shiroma et al. [1] (Brazil)	G1: 2% lidocaine gel	NS	G1: 0	G1: 26.3 ± 16.8	G1: 26.3	G1: MI
	G2: 3.5% lidocaine gel		G2: 0	G2: 20.8 ± 13.5	G2: 20.8	G2: MI
	G3: 5% lidocaine gel		G3: 0	G3: 20.0 ± 16.5	G3: 20.0	G3: MI
	G4: 8% lidocaine gel		G4: 0	G4: 19.3 ± 14.0	G4: 19.3	G4: MI
	G5: 12% lidocaine gel		G5: 0	G5: 18.3 ± 13.5	G5: 18.3	G5: MI
Andrade et al. [6] (Brazil)	G1: proparacaine	SC injection of 2% lidocaine was most effective, with clinical significance (>12 mm)	NA	G1: 32 ± 17	NA	G1: MI
	G2: proparacaine + SC injection of 2% lidocaine			G2: 10 ± 10		G2: MI
	G3: 2% lidocaine gel			G3: 10 ± 11 *p* = 0.02		G3: MI
Kumar et al. [15] (UK)	G1: 0.5% proximetacaine	NS	G1: 6.0 ± 0.63	G1: 8.7 ± 0.83	G1: 14.7	G1: MI
	G2: SC injection of 2% lidocaine		G2: 6.0 ± 0.96	G2: 9.3 ± 0.96 *p* = 0.84	G2: 15.3	G2: MI
Davis et al. [2] (USA)	GA: 0.5% topical proparacaine	NS	NA	G1: 17.8 ± 14.4	NA	G1: MI
	GB: topical proparacaine + 4% lidocaine pledget			G2: 17.5 ± 14.6		G2: MI
	GC: 3.5% lidocaine gel			G3: 14.8 ± 15.8 *p* = 0.38		G3: MI
Rifkin and Schaal [5] (USA)	G1: 0.5% TetraVisc	Statistically different, but not clinically significant	NA	G1: 33.9 ± 22.6	NA	G1: MI
	G2: proparacaine			G2: 31.7 ± 21.8		G2: MI
	G3: tetracaine			G3: 30.5 ± 20.1 *p* < 0.01		G3: MI

*G* group, *MI* mild, *S* significant, *NS* not significant, *NA* not available

### Clinically meaningful difference in pain scores

Pain scores reflecting clinically significant anesthetic techniques for IVI are shown in Table 2. Three studies [5, 6, 14] showed a statistically significant difference in pain scores between different anesthetic techniques. However, only one study [6] presented a clinically significant pain score change of at least 12 mm in a 100 mm scale.

Andrade et al. [6] compared the anesthetic effectiveness of topical proparacaine drops alone, proparacaine + subconjunctival injection of 2% lidocaine, and 2% lidocaine gel in 92 patients. A subconjunctival injection of 2% lidocaine was most effective in preventing pain compared with the two other groups. Difference in pain score between subconjuctival injection (10 mm) or lidocaine gel (10 mm) versus proparacaine (32 mm) was 22 mm. These differences were considered clinically meaningful.

## Discussion

Our systematic review of the literature revealed mild pain in studies about IVI of antiangiogenic agents and steroids, regardless of anesthetic technique. With moderate level of evidence, no single anesthetic technique could be defined as the best option for IVI.

Comparison of anesthetic techniques requires the use of appropriate pain assessment tools [17]. Pain VAS and numerical rating scales (NRS) are considered reliable to evaluate the efficacy of anesthetic or analgesic treatments [8]. Some trials also use four-point verbal categorical pain scales (VRS) to assess discomfort, although VAS and NRS are considered superior to VRS [9]. VAS and NRS have similar sensitivity, and the choice between them is subjective. To provide a meaningful interpretation of pain scale scores, we converted pain scores into Jensen’s levels of pain [8]. All included studies presented mild discomfort during IVI, regardless of anesthetic technique. In this setting, even an effective treatment would show only a small change in pain intensity, and a comparison among anesthetic techniques would probably exhibit low sensitivity [17]. Of the eight included studies, three studies [5, 6, 14] showed a statistically significant difference in VAS or NRS pain scores between different anesthetic techniques. However, only one study [6] found a clinically significant pain score change of at least 12 mm in a 100 mm VAS or NRS scale. In that study, Andrade et al. [6] concluded that a subconjunctival injection of 2% lidocaine was most effective in preventing pain compared to lidocaine gel or proparacaine drops. However, Andrade et al. [6] did not describe the level of discomfort during IVI under subconjunctival anesthesia. Moreover, Kumar et al. [15] failed to observe similar benefits of the subconjunctival approach. The penetration and duration of gel is an important point in topical anesthesia. Lidocaine is absorbed extensively following mucosal intramuscular, rectal, transdermal, and inhalation pathways [18], studies showed the anesthesia with lidocaine 3.5% gel was achieved within 5 min of application in 92% of the subjects [19].

Rodrigues et al. demonstrated that smaller gauge needles 30-G induced less pain than 26-G. In other study published by van Asten et al. comparing ultrathin 33-G needles or 30-G needle concluded that 33-G needle did not result in lower IVI pain (*p* = 0.758), but tended to cause less vitreal reflux (*p* = 0.054) and may limit scleral damage [20, 21].

Rifkin and Schaal published a study evaluating patients’ during intravitreal injection, under topical anesthesia, and observed factors that could influence pain: improved vision from previous injection, female sex, and age >65 years and number of injections, where pain scores decreased with each consecutive injection. [5] However, in a study published by Moisseiev et al., did not confirm these correlation. They also evaluated the injection site (quadrant), number of injections, presence of diabetes mellitus, and lens status. On analysis of injection location by quadrants, such a trend existed toward less pain in the inferonasal quadrant [22].

A meta-analysis was not considered feasible due to high study heterogeneity. This lack of homogeneity was related to the many different anesthetic techniques compared in each study. In addition, some authors combined the discomfort associated with the anesthetic procedure itself to the pain score, while others did not.

Surveys of retina specialists in different countries demonstrated a predominance of topical anesthetic eye drops for IVI [5, 23]. Indeed, in all studies included in this systematic review, proparacaine or tetracaine drops were used; eye drops were either combined with other types of anesthetics to relieve the discomfort caused by dilating drops or povidone iodine drops, or as a single anesthetic choice.

Although eye drops were effective in all selected studies, we could not define a single local anesthetic technique as the gold standard for IVI. Individual preferences, either from the ophthalmologist or the patient, should guide the choice of topical anesthesia. Perhaps, in patients with high sensitivity to pain, a subconjunctival injection of 2% lidocaine could be an option. Considering only pain scores, the low level of discomfort associated to the procedure makes comparing anesthetic techniques a challenge due to low sensitivity. Future studies should focus on side effects of drugs or patient rejection of a specific technique rather than preference.

One of the limitations of this systematic review is the heterogeneity of the included studies, with no synthesis of individual results. Ranking of pain scores according to Jensen’s classification is reliable even in the absence of a meta-analysis, but neither it derives from a statistical tool nor it generates a single median score for each technique. Nonetheless, only three of the included studies were judged to be at low risk of bias based on random sequence generation.

The aim of this review was to evaluate the effectiveness of different local anesthetic techniques for IVI. We considered the pain only at the moment of injection. Chemical keratitis could cause discomfort, even hours after the injection, caused by anesthetics or mydriatic drops, lidocaine gel or PVPI. It usually solved before the first postoperative day.

In conclusion, patient pain was mild in all studies regardless of anesthetic technique. With moderate level of evidence, no single anesthetic technique could be defined as the best option for IVI. Although complete pain relief was not attained, a subconjunctival injection of 2% lidocaine could be an option for highly sensitive patients. Mild pain suggests future studies should focus on side effects or rejection of a specific technique rather than preference.

## Additional files

**Additional file 1: Table S1.**. Database search strategy.

**Additional file 2: Table S2.** Excluded articles with reasons for exclusion.
