# Supplementary material for: Patient pain during intravitreal injections under topical anesthesia: a systematic review
Source: Int J Retina Vitreous. 2017 Jul 3;3:23. doi: 10.1186/s40942-017-0076-9 (PMC5494853; doi:10.1186/s40942-017-0076-9)
Supplement: Supplementary file 1 — Additional file 1: Table S1.. Database search strategy. [file 40942_2017_76_MOESM1_ESM.pdf]

S1 Table. Database search strategy.

**Databases**	**Search Strategy**
Cochrane, LILACS, PubMed, Scopus, Web of Science, Google Scholar	(INTRAVITREAL INJECTION) AND (ANESTHESIA OR LIDOCAINE OR PROXYMETACAINE OR PROPARACAINE OR TETRACAINE OR TOPICAL OR GEL) AND (“ANTIANGIOGENIC AGENTS” OR BEVACIZUMAB OR RANIBIZUMAB OR AFLIBERCEPT OR TRIAMCINOLONE OR CORTICOIDS OR DEXAMETHASONE)
