# Supplementary material for: Patient pain during intravitreal injections under topical anesthesia: a systematic review
Source: Int J Retina Vitreous. 2017 Jul 3;3:23. doi: 10.1186/s40942-017-0076-9 (PMC5494853; doi:10.1186/s40942-017-0076-9)
Supplement: Supplementary file 2 — Additional file 2: Table S2. Excluded articles with reasons for exclusion. [file 40942_2017_76_MOESM2_ESM.docx]

**S2 Table.** Excluded articles with reasons for exclusion

| Author, Reference | Reason for Exclusion |
| --- | --- |
| Cintra et al. [1] | 8 |
| Cohen et al. [2] | 6 |
| Lyall et al. [3]  Hernandez-Siman et al. [4]  LaHood et al. [5]  Kaderli & Avci [6]  Gregori et al. [7]  Sanabria et al. [8]  Tewari et al. [9]  Page & Fraunfelder [10]  Tailor et al. [11]  Moisseiev et al. [12]  Moisseiev et al. [13]  Karabas et al. [14]  Kozak et al. [15] | 2  2  2  6  7  7  2  2  2  5  5  5  5 |

Key to “reason for exclusion”: (1) studies conducted in infants (0–18 years); (2) reviews, letters, conference abstracts, and editorials; (3) studies including sedation or general anesthetics for intravitreal injection; (4) studies including intravitreal injection of medications other than antiangiogenic agents or steroids; (5) non-randomized clinical trials; (6) studies using a pain scale other than an analogue (visual or oral) scale ranging from 0 to 100 or 0 to 10; (7) studies not evaluating pain at the moment of injection; and (8) studies not using mean as the measure of central tendency for pain score.

References

[1] Cintra LP, Lucena LR, Da Silva JA, Costa RA, Scott IU, Jorge R. Comparative study of analgesic effectiveness using three different anesthetic techniques for intravitreal injection of bevacizumab. Ophthalmic Surg Lasers Imaging. 2009/02/12 ed. Ribeirao Preto School of Medicine, Sao Paulo, Brazil.; 2009;40:13–8.

[2] Cohen SM, Billiris-Findlay K, Eichenbaum DA, Pautler SE. Topical lidocaine gel with and without subconjunctival lidocaine injection for intravitreal injection: a within-patient study. Ophthalmic Surg Lasers Imaging Retin. 2014/07/19 ed. 2014;45(4):306–10.

[3] Lyall DAM, Tey A, Foot B, Roxburgh STD, Virdi M, Robertson C, et al. Subconjunctival anaesthesia for intravitreal injections Reply. Eye [Internet]. 2013;27(9):1110.

[4] Hernandez-Siman J, Marra K, Arroyo J. Sub-conjunctival lidocaine injection before intravitreal injection. Surv Ophthalmol. 2014/12/03 ed. Boston, Massachusetts.; 2014;59(6):672–3.

[5] LaHood BR, Sherwood D, Suter A. Comparative assessment of the effectiveness of anaesthesia for intravitreal bevacizumab injection. Clin Exp Ophthalmol [Internet]. 2011;39(2):184–5.

[6] Kaderli B, Avci R. Comparison of topical and subconjunctival anesthesia in intravitreal injection administrations. Eur J Ophthalmol. 2006/10/25 ed. Department of Ophthalmology, Uludag University Hospital, Bursa, Turkey. drkaderli@yahoo.com; 2006;16(5):718–21.

[7] Gregori NZ, Weiss MJ, Goldhardt R, Schiffman JC, Vega E, Mattis C-A, et al. Randomized clinical trial of two anesthetic techniques for intravitreal injections: 4% liquid lidocaine on cotton swabs versus 3.5% lidocaine gel. Expert Opin Drug Deliv [Internet]. 2012;9(7):735–41.

[8] Sanabria MR, Montero JA, Losada M V, Fernandez-Munoz M, Galindo A, Fernandez I, et al. Ocular pain after intravitreal injection. Curr Eye Res. 2013/01/22 ed. Ophthalmology Unit, Palencia Hospital Complex (CAPA), Palencia, Spain.; 2013;38(2):278–82.

[9] Tewari A, Shah GK, Dhalla MS, Blinder KJ. Surface anesthesia for office-based retinal procedures. Retin J Retin Vitr Dis [Internet]. 2007;27(6):804–5.

[10] Page MA, Fraunfelder FW. Safety, efficacy, and patient acceptability of lidocaine hydrochloride ophthalmic gel as a topical ocular anesthetic for use in ophthalmic procedures. Clin Ophthalmol. 2009/11/10 ed. Department of Ophthalmology (Casey Eye Institute), Oregon Health and Science University, Portland, OR, USA.; 2009;3:601–9.

[11] Tailor R, Beasley R, Yang Y, Narendran N. Evaluation of patients’ experiences at different stages of the intravitreal injection procedure - what can be improved? Clin Ophthalmol. 2011/11/10 ed. Wolverhampton and Midland Counties Eye Infirmary, New Cross Hospital, Wolverhampton, UK.; 2011;5:1499–502.

[12] Moisseiev E, Regenbogen M, Bartfeld Y, Barak A. Evaluation of Pain in Intravitreal Bevacizumab Injections. Curr Eye Res [Internet]. 2012;37(9):813–7.

[13] Moisseiev E, Regenbogen M, Rabinovitch T, Barak A, Loewenstein A, Goldstein M. Evaluation of pain during intravitreal Ozurdex injections vs intravitreal bevacizumab injections. Eye [Internet]. 2014;28(8):980–5.

[14] Karabaş VL, Özkan B, Koçer ÇA, Altntaş Ö, Pirhan D, Yüksel N. Comparison of two anesthetic methods for intravitreal Ozurdex injection. J Ophthalmol [Internet]. Ophthalmology Department, Faculty of Medicine, Kocaeli University, Kocaeli, Turkey; 2015;2015.

[15] Kozak I, Cheng LY, Freeman WR. Lidocaine gel anesthesia for intravitreal drug administration. Retin J Retin Vitr Dis [Internet]. 2005;25:994–8.
